# The mutational landscape of atypical chronic myeloid leukemia

**DOI:** 10.17179/excli2019-1246

**Published:** 2019-05-16

**Authors:** Stephen E. Langabeer

**Affiliations:** 1 Cancer Molecular Diagnostics, St. James's Hospital, Dublin, Ireland

## ⁯

***Dear Editor,***

Atypical chronic myeloid leukemia (aCML) is a rare haematological malignancy classified as a myelodysplastic/myeloproliferative neoplasm (MDS/MPN). Diagnostic criteria include leucocytosis with left shift, dysgranulopoiesis, minimal basophila and monocytosis, a hypercellular bone marrow, less than 20 % myeloblasts in the blood or bone marrow, and absence of the distinct molecular abnormalities of classical MPN (Arber et al., 2016[1]). Until recently, treatment for aCML has been largely based on those agents used for either MDS or MPN type with varying degrees of success (Gotlib, 2017[5]). While haematopoietic allogeneic stem cell transplantation remains the only curative therapy, it is limited to those aCML patients eligible for such a procedure (Onida et al., 2017[11]). In other myeloid malignancies, targeted sequencing has identified recurrently mutated genes providing both insight into disease pathogenesis and helped to unmask therapeutically actionable molecular abnormalities. 

**Figure 1:**
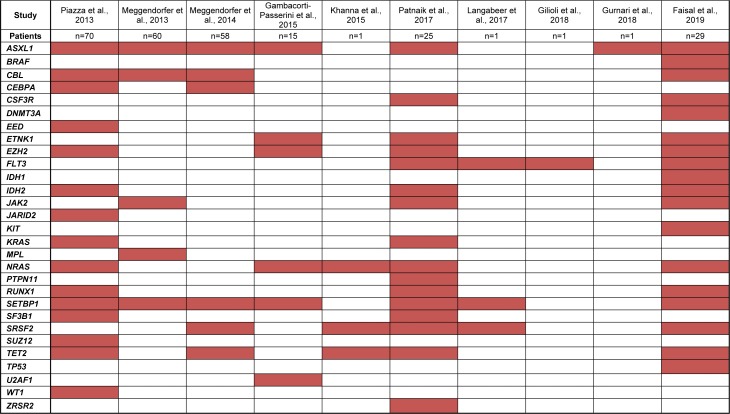
Mutated myeloid malignancy-associated genes (red shading) by targeted sequencing in aCML patients

Several studies have investigated the mutation profile of sizeable cohorts of aCML patients in chronic and blast crisis phases by targeted sequencing of recurrently mutated genes of myeloid malignancies. However, in order to comprehensively survey the current mutational landscape of aCML, these studies together with smaller series and case reports adopting such targeted sequencing approaches in the literature, are summarised in Figure 1(Fig. 1) (References in Figure 1: Piazza et al., 2013[13]; Meggendorfer et al., 2013[9]; Meggendorfer et al., 2014[10]; Gambacorti-Passerini et al., 2015[3]; Khanna et al., 2015[7]; Patnaik et al., 2017[12]; Langabeer et al., 2017[8]; Gilioli et al., 2018[4]; Gurnari et al., 2018[6]; Faisal et al., 2019[2]). Caveats include the use of evolving classification criteria for aCML, possible duplication of patients in two or more studies, and differences in the genes covered by independent targeted sequencing approaches.

From this summary, the most frequently mutated genes in aCML patients appear to be *ASXL1*, *NRAS*, *SETBP1*, *SRSF2* and *TET2*. In addition to broad cytoreductive and supportive measures (Schwartz and Mascarenhas, 2019[14]), clinical responses with inhibitors of mutated *NRAS* and *FLT3* have been observed in single cases validating this individualized approach (Khanna et al., 2015[7]; Langabeer et al., 2017[8]).

Due to the lack of a standard of care combined with the clinical and genetic heterogeneity of this malignancy, a personalized prognostic and therapeutic direction to aCML management is anticipated, facilitated by the progressively fundamental role of targeted mutation profiling.

## Conflict of interest

The author declares no conflict of interest.
